# Cataract Surgery in nAMD Patients Receiving Intravitreal Aflibercept Injections

**DOI:** 10.3390/jcm13133832

**Published:** 2024-06-29

**Authors:** Małgorzata Seredyka-Burduk, Slawomir Liberski, Grażyna Malukiewicz, Jarosław Kocięcki, Bartlomiej J. Kaluzny

**Affiliations:** 1 Division of Ophthalmology and Optometry, Department of Ophthalmology, Collegium Medicum, Nicolaus Copernicus University, Kornela Ujejskiego 75 Street, 85-168 Bydgoszcz, Poland; 2 Department of Ophthalmology, Poznan University of Medical Sciences, Augustyna Szamarzewskiego 84 Street, 61-848 Poznań, Poland

**Keywords:** cataract, neovascular age-related macular degeneration, anti-VEGF agents, phacoemulsification

## Abstract

**Background:** To evaluate functional and anatomical outcomesof cataract surgery in neovascular age-related macular neovascularization (nAMD) eyes receiving anti-vascular endothelial growth factor (anti-VEGF) intravitreal injections in modified pro re nata (PRN) regimen. **Materials and Methods:** Sixty eyes of 60 nAMD patients, including 41 women (68.3%) and 19 men (31.7%) in an average age of 77.35 ± 6.41 years, under treatment with intravitreal aflibercept injections in modified PRN regimen with no signs of macular neovascularization (MNV) activity during two consecutive visits were included in this prospective, observational study. Best-corrected visual acuity (BCVA), central retinal thickness (CRT), as well as the number of anti-VEGF injections were monitored six months before and after phacoemulsification with intraocular lens (IOL) implantation. Further, the change of the abovementioned parameters was assessed during the six-month follow-up period for CRT and the number of injections, while the BCVA was monitored for 54 months. **Results:** BCVA measured on the first day after surgery (0.17 ± 0.19 logMAR) as well as in the six-month post-surgery (0.13 ± 0.16 logMAR) significantly improved compared to preop values (0.42 ± 0.20 logMAR). BCVA remains stable during the observational period. We found that both differences were statistically significant (*p* < 0.01). The mean CRT and the mean number of injections did not differ between the six-month pre- and post-surgical periods. **Conclusions:** We showed the beneficial effect of phacoemulsification in nAMD patients treated with anti-VEGF agents on visual outcomes in the short and long term. Cataract surgery in nAMD eyes treated with anti-VEGF injections does not increase the frequency of anti-VEGF injections and does not cause deterioration of the macular status.

## 1. Introduction

Vision loss or visual impairment is estimated to affect approximately 20 percent of the elderly population and is one of the main factors affecting the quality of life, contributing to an increased risk of depressive disorders, poorer physical health, and a higher mortality rate [1,2]. Cataract and age-related macular degeneration (AMD) are two of the four most common age-related eye diseases responsible for blindness in 15 and 8 million individuals aged over 50 worldwide, respectively [3]. Due to the common risk factors for both conditions, including age, smoking, obesity, cardiovascular diseases, and increased exposure to ultraviolet (UV) radiation, the high co-occurrence of AMD and cataract is observed [4,5,6]. The comorbidity of AMD and cataract in the visually impaired population is present in 13.7% of individuals over 65 [7], while in a recent study, the optical coherence tomography (OCT) examination revealed AMD signs in 20% of patients over 50 years qualified for cataract surgery [8]. In further years, the comorbidity of cataract and AMD is presumed to increase due to the aging of societies and the extension of life expectancy [7]. Therefore, unfavorable epidemiological forecasts made both diseases of crucial public health concern in recent years [9].

The potential impact of cataract surgery on the risk of AMD progression is still controversial, and previous research showed inconsistent outcomes [2,4,7,9]. It has been postulated that cataract removal might cause the intensification of AMD activity and lead to the progression of dry AMD to nAMD. The risk of previously diagnosed nAMD exacerbation and weaker anti-VEGF therapy response due to surgery-induced pro-inflammatory mediators release and intraoperative changes in intraocular pressure (IOP) stimulating the development of neovascular lesions [10,11], which may result in submacular exudates and hemorrhages formation in the postoperative period was also postulated [5]. Other concerns regard the appropriateness of cataract surgery in nAMD patients in perspective of uncertain prognosis of vision improvement. On the other hand, cataract progression leads to further visual acuity deterioration of nAMD eyes and limits both the benefits of anti-VEGF treatment and hinders proper examination of the macula during fundoscopy [10,11]. Therefore, the decision regarding cataract surgery in individuals with coexisting AMD is still debatable, and more than 50% of surgeons in the UK rely on personal experience during decision-making [12].

Therefore, to clarify this issue, we assessed the impact of cataract surgery in nAMD patients on visual and anatomical outcomes, as well as the number of necessary anti-VEGF injections. Understanding the effect of cataract surgery on these parameters in nAMD individuals will help to obtain a consensus on the appropriate management in this group of patients.

## 2. Materials and Methods

### 2.1. Study Design

This prospective observational study was performed at the Department of Ophthalmology of the Nicolaus Copernicus University in Toruń, Poland. Sixty nAMD patients treated with intravitreal injections of aflibercept (Regeneron Inc., Tarrytown, NY, US, and Bayer HealthCare Inc., Berlin, Germany) who underwent small-incision phacoemulsification cataract surgery with subsequent intraocular lens (IOL) implantation were enrolled in the study. Only one eye of each participant was enrolled.

Participants received intravitreal aflibercept injections from a publicly funded neovascular nAMD drug program. According to the treatment schedule, each patient had monthly control visits with an assessment for eligibility for further injection depending on the presence of disease activity signs. The Coordinating Team for the Treatment of Retinal Diseases (Military Medical Institute, Warsaw, Poland) qualified each patient to participate in the drug program. Following the criteria specified by the Polish Ministry of Health, patients over 45 years with active CNV involving more than 50% of the macular lesion confirmed by OCT and angiography were suitable for enrolment. In addition, the BCVA must enclose in the range of 0.2–0.8 (decimal), and the total lesion size less than 12 areas of the optic nerve head without dominant hemorrhage, geographic atrophy, and permanent disruption of the foveal structure. Each patient had to give written consent to participate in the program and receive intravitreal injections. The study included non-treatment naive nAMD patients treated with anti-VEGF injections with co-occurring cataract confirmed in the slit lamp examination and BCVA equal to or below 0.6 (decimal) measured with the use of Snellen chart and no concomitant contraindications for cataract surgery. Individuals with CNV type I or II confirmed by fluorescein angiography (AF) were enrolled. The exclusion criteria were the presence of significant, irreversible macular damage manifested as the presence of a dominant subretinal scar, or geographical atrophy (GA). Conditions that may affect the assessment of visual acuity and macular condition, such as pathological corneal changes, the presence of high myopia, current or past retinal vascular occlusion, vitreous hemorrhage, advanced glaucoma or uncontrolled intraocular pressure, diabetic retinopathy, present macular hemorrhage, as well as current or past retinal detachment were exclusion criteria.

In addition to a BCVA of 0.6 (decimal) or less and the confirmed presence of lens opacity in the slit lamp examination, the eligibility condition for cataract surgery was the absence of signs of neovascular membrane activity during two consecutive follow-up visits. Each patient’s management schedule was designed to ensure that the cataract removal procedure did not interfere with the anti-VEGF injection regimen.

Written informed consent was collected from participants after being introduced to the study principles in written and oral form prior to inclusion. This study was approved by the Bioethics Committee of the Nicolaus Copernicus University in Toruń at Collegium Medicum in Bydgoszcz (KB 107/2022), Poland, and was in accordance with the principles included in the Declaration of Helsinki.

### 2.2. Pre- and Postoperative Monitoring of Participants and Data Collection

BCVA was measured at six monthly follow-up visits before cataract surgery. BCVA was also assessed on the day of surgery, on the first day after surgery, and at six monthly follow-up visits after phacoemulsification. Further BCVA measurements were prolonged up to 54 months, and during this period, visual acuity was examined at least every four months for each participant. Visual acuity was assessed with the use of Snellen charts and then was converted to the logMAR. Based on 3D macular scans, CRT was determined using the SS-OCT device (DRI OCT Triton, Topcon, Inc., Tokyo, Japan). Macular CRT scans were performed at six-monthly visits before and after cataract surgery.

We also evaluated the effect of cataract surgery on the frequency of aflibercept injections in modified pro re nata (PRN) regimen in the postoperative six months as well as total period and compared it with the corresponding periods before surgery.

### 2.3. Intravitreal Injections of Aflibercept

Intravitreal injections of aflibercept were administered in the operating room according to the standard procedure for intravitreal injections.

Patients were treated according to the modified PRN regimen, after a loading phase of three-monthly intravitreal injections followed by monthly observations during control visits. The criteria for treatment resumption included detecting disease activity such as the appearance of any amount of SRF or IRF on the macular OCT scan, subretinal hemorrhages observed in the macular region, or visual loss. When the absence offluid was observed at one visit, the injection was given, while the absence of fluid at two consecutive visits indicated a non-injection monitoring visit.

### 2.4. Cataract Extraction Procedure and Postoperative Care

All patients underwent cataract surgery by phacoemulsification by making a 2.6mm incision and using the divide-and-conquer technique. Manual capsulorhexis was performed, and the cortex was removed by irrigation and aspiration. The foldable, hydrophobic acrylic lens with an ultraviolet (UV) light filter was implanted in each patient—AcrySof UV-filtering IOL (SA60AT, Alcon Laboratories Inc., Fort Worth, TX, USA). The Centurion Vision System (Alcon Laboratories Inc., Geneva, Switzerland) was used to perform the surgical procedures. The same experienced surgeon performed all cataract surgeries.

After the procedure, all patients received topical treatment into the operated eye according to the same schedule, including antibiotic fluoroquinolone drops for two weeks (five times a day), as well as four weeks of therapy with a non-steroidal anti-inflammatory drug (four times a day) and a steroid (six times daily for one week with a subsequent dose reduction).

There were no intraoperative complications in the study group. Similarly, none of the study participants had ophthalmic complications associated with cataract surgery in the early postoperative period (examination on the first day after surgery) and during the six-month follow-up.

### 2.5. Sample Size Calculation

The sample size was calculated using the results of Brant et al. [13]. Based on the cited study, we found that a sample size of 26–104 eyes was required to detect a significant difference in visual acuity at a significance level of *p* < 0.05 and a power of 80%, with an assumed sampling error of 0.1 and 0.05, respectively. These calculations are consistent with the sample sizes of previous studies in this area [10,11,14,15,16,17,18,19,20,21,22].

### 2.6. Statistical Analysis

Statistical analysis of the data was carried out in the program Statistica (Data Analysis Software System) ver. 13.1 (StatSoft, Inc., St Tulsa, OK, USA). The analysis was based on the Friedman ANOVA test, the Spearman rank order test, and the Wilcoxon and U Mann-Whitney pair order tests.The choice of tests from the group of non-parametric tests was conditioned, among others, by failure to meet the basic assumptions of parametric tests, i.e., compliance of the distributions of the studied variables with the normal distribution or uniformity of variance. The compatibility of the distributions of the studied variables with the normal distribution was verified by the Shapiro-Wilk W test. At the same time, the uniformity of variance was assessed by the Levene test. For the purposes of this work, in the figures, one month represents 4.35 weeks. The level of statistical significance was *p* < 0.05.

## 3. Results

### 3.1. Demographic Data of the Study Population

Sixty nAMD patients, including 41 women (68.3%) and 19 men (31.7%) with an average age of 77.35 ± 6.41 years were enrolled. The mean time from diagnosis of nAMD to initiation of anti-VEGF treatment in the study group was 2.95 ± 1.95 weeks, while the mean time from the start of anti-VEGF therapy to cataract surgery was 28.98 ± 20.89 months (Table 1).

### 3.2. Visual Acuity

**Figure 1:**
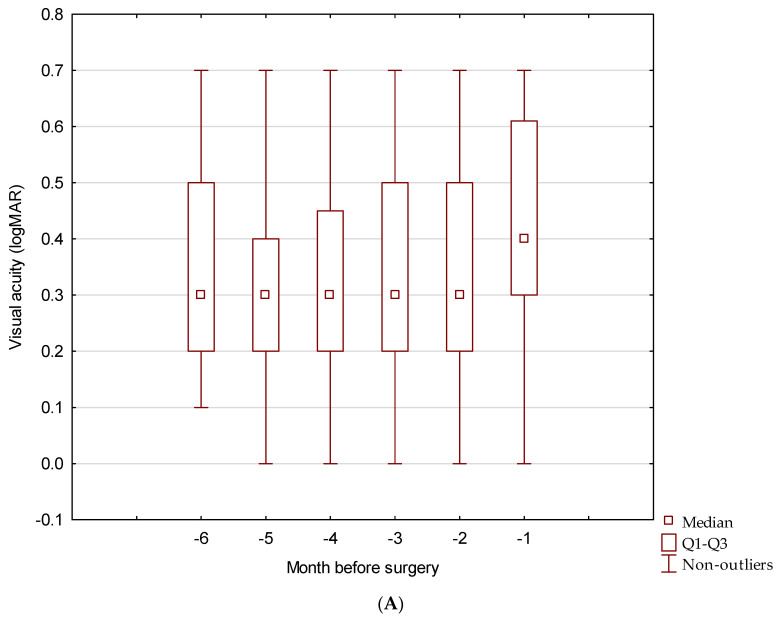

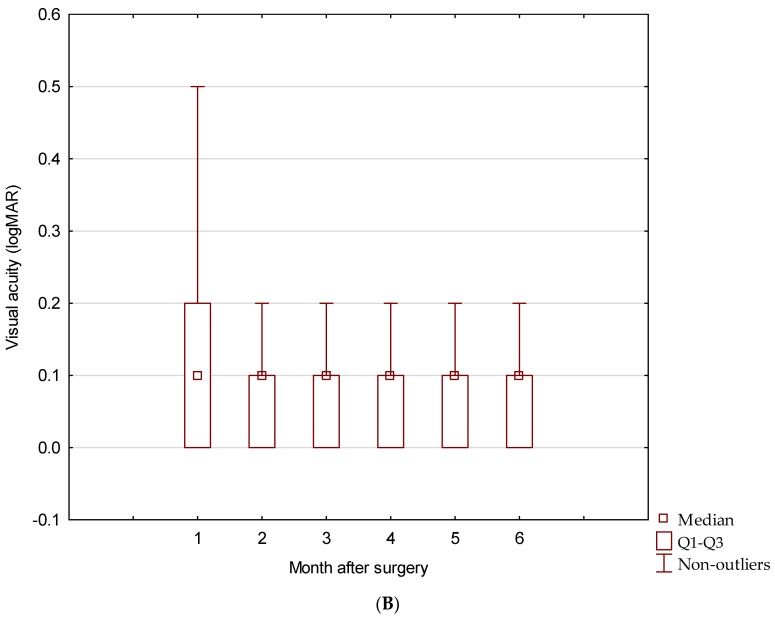
BCVA of nAMD patients six-month before cataract surgery (**A**), and during six-month follow-up after cataract surgery (**B**).

**Figure 2:**
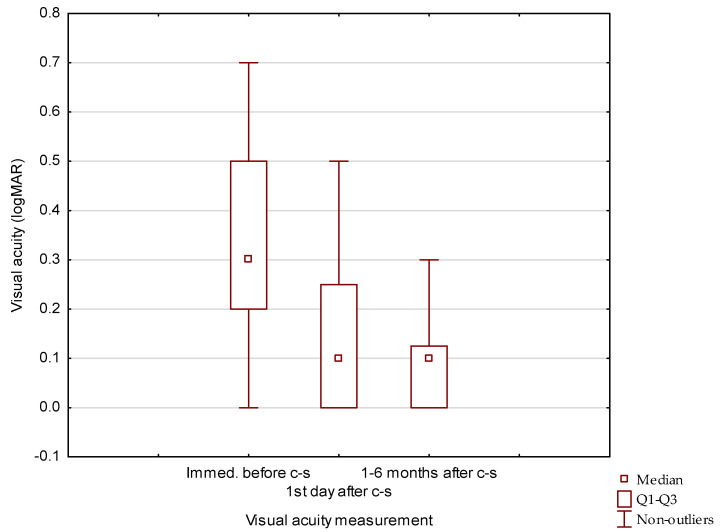
Comparison of BCVA measurements immediately before cataract surgery (c-s) and at the first day and the mean of monthly measurements during six months follow up after cataract surgery (c-s).

Best-corrected visual acuity (BCVA) in the study group was monitored at monthly follow-up visits for six consecutive months before the phacoemulsification. During the preoperative period, BCVA differed significantly in six consecutive measurements (*p* < 0.001) (Figure 1B) (Table 2). The average BCVA, six months before surgery, and one month before surgery was 0.48 ± 0.19 and 0.42 ± 20 logMAR, respectively (Figure 2).

The BCVA measured during the control visit on the first day after cataract removal (0.17 ± 0.19 logMAR) significantly improved compared to the BCVA observed on the day of surgery (0.34 ± 0.22). In the first month after surgery, the mean BCVA was 0.13 ± 0.17, and it remained stable during monthly visits of the six-month post-surgery follow-up with mean BCVA (0.14 ± 0.19) (Figure 1) (Table 3). We found that both differences were statistically significant (*p* < 0.01) (Figure 2). Further observation showed that BCVA remained stable during the next 42 months and was 0.17 ± 0.18 and 0.20 ± 0.18 at 19–24 and 37–42 months, respectively. After 42 months, variability in BCVA measurements was observed. The average BCVA at 6-month intervals was 0.25 ± 0.21 (months 43–48), and 0.21 ± 0.17 (months 49–54) (Table 4).

### 3.3. Central Retinal Thickness

**Figure 3:**
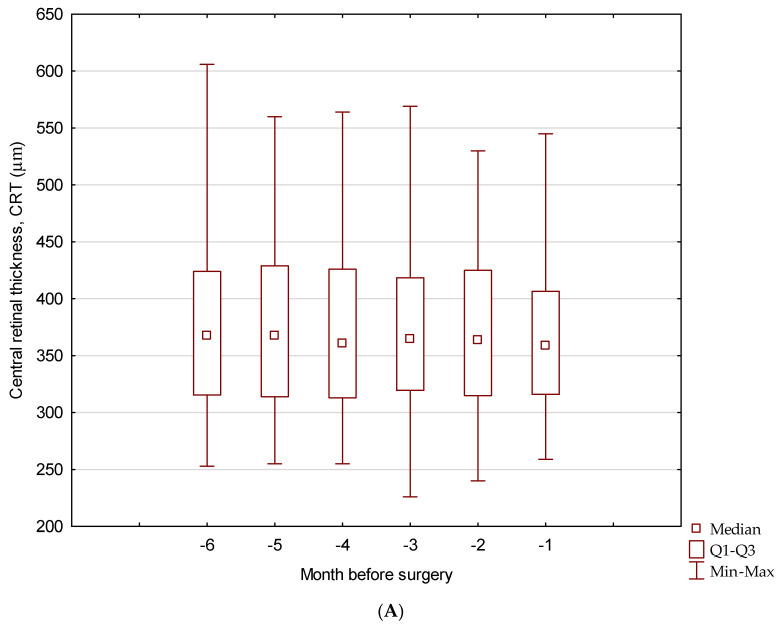

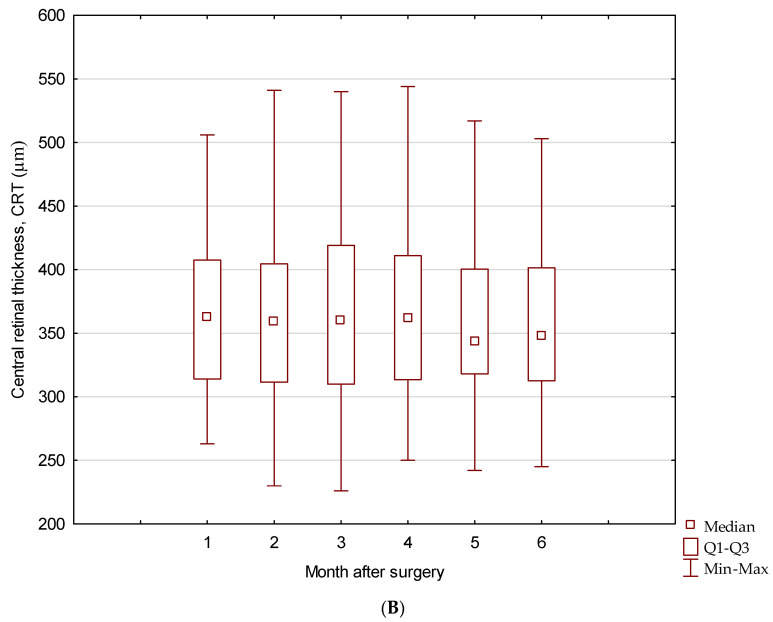
Box-plot chart showing comparison of mean CRT value during pre- (**A**) and post-operative (**B**) six-month follow-up.

The mean central retinal thickness (CRT) was stable in six monthly measurements before (*p* = 0.358) and after phacoemulsification (*p* = 0.192). Comparing of the mean CRT over the six months before and after surgery showed no marked differences (*p* = 0.216). The CRT values after surgery were slightly lower (363.61 µm) than those before the surgery (373.85 µm) (Figure 3).

**Figure 4:**
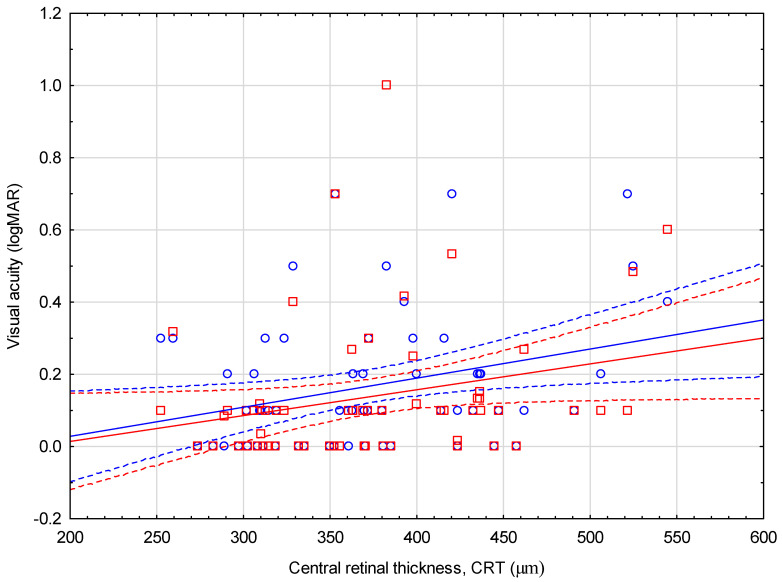
Correlation between mean six-month pre-operative CRT and BCVA at day one after cataract surgery. Abbreviations: BCVA, Best-corrected visual acuity (Snellen charts); CRT, central retinal thickness. Dots—BCVA at day one after cataract surgery; Squares—mean six-month pre-operative CRT.

A negative correlation was shown between the mean CRT from the six-month pre-surgical period and BCVA measured on a control visit one day after surgery (r = −0.27; *p* = 0.036) and the average BCVA from six months after cataract removal (r = −0.32; *p* = 0.013) (Figure 4). Similar correlations were not confirmed for the post-surgery CRT and BCVA measurements (*p* > 0.05) (Table 5).

### 3.4. Pre- and Postoperative Frequency of Intravitreal Injections

In the six-month before surgery period, each patient received an average of 2.9 ± 0.71 injections of aflibercept, while during six-month post-surgery, it averaged 2.75 ± 0.77 (*p* < 0.001).

Further, the time intervals between injections in the total time and six-month period before and after phacoemulsification were compared, and a difference was found for the total number of injections, where the time between injections was prolonged in the postoperative period (*p* = 0.07) (Table 6). The last injection before cataract surgery was performed 4.46 ± 2.27 weeks before cataract surgery, while the first injection after surgery was administered within 4.70 ± 2.81 weeks. There was no correlation between the change in visual acuity before and six months after surgery and the time to the first injection after surgery (r= −0.11; *p* = 0.4).

## 4. Discussion

Although cataract surgery in dry AMD patients is relatively safe, the decision to perform surgery in eyes with nAMD is still controversial [23]. Progress in nAMD treatment prior to the introduction of anti-VEGF injections and advancements in cataract surgery techniques through the introduction of small-incision phacoemulsification associated with less postoperative inflammation, prompter regeneration, and less phototoxicity has allowed better management of eyes with co-occurring cataract and nAMD [23]. Nevertheless, despite ongoing advances in treating cataract and nAMD, the final visual acuity after cataract surgery in nAMD eyes is lower compared to healthy eyes. The results of the Report No. 5 of the AREDS 2 study showed that visual outcomes after cataract surgery in nAMD individuals are directly proportionally dependent on the severity of AMD before surgery. In eyes with advanced AMD (GA or nAMD), BCVA after seven months was almost twice lower than in eyes with mild AMD, according to the AREDS AMD severity scale (AAS) [24]. In contrast, 10 and 20 years of observational studies revealed that AMD is the most common factor leading to significant loss of visual acuity in eyes undergoing cataract surgery [25,26]. Although cataract surgery does not usually improve central vision in eyes with advanced AMD involving the presence of GA or fibrous scarring causing permanent damage to the macular structure, performing phacoemulsification in patients with nAMD may contribute to a more satisfactory contrast, as well as enhanced color and peripheral vision. While in nAMD eyes with lower severity of pathological changes in the macula, cataract surgery is also beneficial in terms of improving central vision [27].

Our results demonstrated a significant improvement in BCVA on the first day after surgery, remaining stable during monthly follow-up visits over the following 48 months of the follow-up period. Previous studies conducted according to differentiated schedules with an observation period of one month to one year also demonstrated an improvement in BCVA of nAMD patients after cataract surgery. Monthly follow-up of patients with concomitant choroidal neovascularization (CNV) on the background of nAMD undergoing phacoemulsification combined with a single IVB injection revealed a marked improvement in mean BCVA from 20/100 to 20/63 Snellen acuity [28]. In the study by Saraf et al., an improvement in visual acuity in nAMD patients undergoing cataract surgery both before surgery (visual gain 0.23 ± 0.65 logMAR) and from controls in unoperated patients (visual gain 0.11 ± 0.59 logMAR) has been observed after three-month follow-up [11]. Post-hoc analysis of ANCHOR and MARINA studies demonstrated that after cataract surgery BCVA in nAMD eyes treated with intravitreal ranibizumab (IVR) increased by an average of 10.4 ± 3.4 letters during three months post-surgery control visit [23]. In studies with monitoring extended to 12 months beneficial outcomes of cataract surgery on BCVA in nAMD eyes treated with anti-VEGF agents have also been observed. In a retrospective study on a larger group of nAMD patients, Tang et al. revealed marked BCVA improvement from 59 ± 12 to 66 ± 15 ETDRS letters during the six-month observational period [21]_._ Similar evidence has been reported by Lee et al. The cited study revealed marked improvement in the BCVA of nAMD patients after cataract surgery after one month and six months compared to the study before surgery [29]. Karesuvo et al. proved BCVA gain both during the first postoperative examination (0.39 ± 0.40 logMAR) and one year after surgery in nAMD patients undergoing phacoemulsification (0.33 ± 0.34 logMAR) compared to baseline (0.70 ± 0.46 logMAR) [10]. Investigation in the nAMD patients with a worse baseline BCVA treated with IVR and undergoing cataract surgery revealed a marked improvement in BCVA (0.94 ± 0.21 vs. 0.48 ± 0.35 logMAR) corresponded to the nAMD patients without the presence of lens opacity where non-significant visual gain was noted (0.77 ± 0.36 vs. 0.49 ± 0.33 logMAR) [20]. Similarly, a visual gain of 8.04 letters after 12 months in the nAMD patients treated with anti-VEGF injections and undergoing phacoemulsification compared to a loss of 1.96 letters in the group of nAMD patients also treated with anti-VEGF agents and characterized by the presence of lens opacity at LOCS II or higher who did not undergo cataract surgery was found [17]. The current study showed that lower 12-month postoperative BCVA in nAMD patients undergoing phacoemulsification was associated with worse baseline visual acuity and ellipsoid zone damage observed in OCT scans before surgery, but not with gender, age, and macular factors (presence of subretinal fluid, SRHM, CNV activity, RPE subillumination, and CST), as well as the duration of nAMD, number of anti-VEGF injections both before and after cataract surgery [30]. Choi et al. showed substantial improvement in BCVA was observed in nAMD patients undergoing phacoemulsification up to 36 months after surgery; however, at follow-up more than three years after surgery, BCVA was not significantly different from the baseline. The analysis showed that nAMD patients with higher BCVA before surgery and shorter duration of disease had a better prognosis for postoperative BCVA [31].

Our extended follow-up to 54 months confirms the long-term beneficial effect of cataract surgery on visual function in nAMD eyes in a longer perspective. In the period of 48–54 months after surgery, BCVA values demonstrated variability; however, the average BCVA was still better than before surgery. Long-term outcomes of the study by Monestam revealed that in the eyes of nAMD, visual improvement after cataract surgery is still present at the end of the 10-year follow-up period [25].

CRT reflects the distance between the ILM layer and the RPE/Bruch membrane measured in the center of the macular fovea [32]. It is known that in nAMD patients, the main factor influencing elevated CRT is retinal fluid leaking through the walls of new, abnormal blood vessels [32]. Therefore, CRT is a well-established and helpful indicator for assessing neovascular membrane activity beneath the macula. In addition, previous studies have shown that changes in this parameter may predict prognosis in nAMD eyes [32]. Interestingly, previous studies have shown that uncomplicated phacoemulsification can increase CRT values in healthy eyes. This phenomenon is probably secondary to the surgery-related release of prostaglandins and pro-inflammatory cytokines. These compounds can penetrate from the anterior to the posterior segment of the eye and cause blood-retina-barrier (BRB) damage, resulting in macular thickening [18]. Usually, the peak of CRT values was observed 6 to 12 weeks after surgery [14,18,22], and in some patients, it may still persist after six months [14].

Our study revealed no significant changes in the mean CRT six months before and after phacoemulsification, suggesting that cataract surgery does not cause the deterioration of the macular condition. Previous studies have revealed conflicting conclusions regarding the effect of cataract surgery on CRT in nAMD patients, exposing a decrease in CRT [10,15,28], an increase in CRT [11], or no marked effect [16,17,20]. In a prospective study, with a one-month follow-up, Furino et al. showed a marked CRT reduction from 353.75 ± 12.50 µm to 275.7 ± 17.3 µm in nAMD eyes after phacoemulsification with simultaneous injection of IVB [28]. Another study demonstrated a decrease in both central macular thickness (CMT) and central subfield macular thickness (CSMT) in a one-year follow-up in nAMD patients undergoing cataract surgery treated with bevacizumab or aflibercept injections [10].

A three-month observation of nAMD eyes underwent phacoemulsification proved a higher incidence of new or worsening of previously observed nAMD activity OCT biomarkers [11], while Grixti et al. found an increase in CMT after one month compared to baseline (203 µm vs. 238 µm). However, an examination three months after phacoemulsification showed a decrease in CMT (212.5 µm), and finally, this parameter was not remarkably changed. In the cited study, among the 30 patients in the drug-free phase before cataract surgery, 27%, 43%, and 57% of patients required re-treatment with anti-VEGF in the first, third, and sixth months of the study, respectively [15]. The recent study by Tang et al. found no significant changes in subretinal fluid (SRF), sub-retinal pigment epithelium fluid (sub-RPE fluid), or CRT after a six-month follow-up period. Interestingly, there was a significant increase in the number of eyes with intraretinal fluid (IRF); however, it was not related to higher treatment intensity or worse BCVA outcomes in this group compared to eyes without an IRF increase [20]. In the other experiments, neither a marked change in CRT [16,17,20] nor an increase in both SRF and intraretinal fluid (IRF) in the macular area in nAMD patients 12 months after cataract surgery has been observed [20]. Discrepancies observed in the baseline CRT values in the abovementioned studies may result from demographic differences between groups and the degree of disease activity and its duration at the moment of surgery. In addition, the use of topical drops dosed in various regimens, especially steroids and NSAIDs, may also affect the reduction of CRT during the postoperative period by inhibiting the inflammatory response of eye tissues [33]. It is worth emphasizing that in some patients, the potential pro-exudative effect of phacoemulsification could be slightly masked by the persistent anti-exudative activity of aflibercept; however, both in our study model and in clinical practice, this impact cannot be fully excluded.

The absence of substantial fluctuations of CRT after cataract surgery is highly desirable regarding the documented association between macular thickness oscillations and worse visual outcomes, as well as a higher percentage of fibrosis and atrophic lesions in the macular region [32,34,35]. We found a negative correlation between the mean six-month pre-operative CRT value and BCVA measured one day after surgery, as well as the meansix-month postoperative BCVA. These results suggest that nAMD eyes with the less disrupted architecture of the macular region have better visual prognoses associated with combined anti-VEGF treatment and phacoemulsification. A similar analysis was performed by Karesvuo et al.; however, the authors did not find any relationship between pre- and post-operative mean central subfield macular thickness (CSMT) and post-operative BCVA [10]. Similarly, Tang et al. did not reveal worse visual outcomes of eyes with new IRF after phacoemulsification [21]. In previous years, the issue of increased retinal exposure to UV radiation and blue light after surgical removal of a cloudy lens with UV-absorbing capabilities has been a significant concern. This problem is particularly relevant in the context of the results of animal studies that have proven the adverse effects of UV radiation on the RPE and photoreceptors. However, with the widespread use of UV and blue-light lenses, this problem has been primarily addressed. Nonetheless, it remains crucial that AMD patients and those at increased risk of AMD have artificial lenses implanted with UV and blue-light-absorbing abilities [17].

The introduction of anti-VEGF agents allowed to stabilize or improve the visual function of eyes affected by CNV. However, many patients require a high frequency of injections to achieve the desired effect, resulting in a high treatment burden and, thus, a reduced quality of life [36]. Current concepts for reducing the number of intravitreal administrations include introducing new anti-VEGF agents with prolonged biological activity and treatment regimens to optimize the treatment schedule. However, properly managing comorbidities such as cataract can also result in a beneficial effect on nAMD patients regarding the treatment burden. Comparison of the number of injections before and after phacoemulsification may reflect changes in disease activity after surgery, as well as allow the assessment of the effect of phacoemulsification on the patient’s burden of anti-VEGF injections.

We found that the number of injections did not differ significantly between six months before and after cataract removal. Previous studies have also shown no significant effect of cataract surgery on the number of injections over six [5,11,15,21,36] and 12 months observation before and after the procedure [10,17,20,30]. Interestingly, Rappaport et al. showed that performing the phacoemulsification procedure in patients with observed CNV activity biomarkers—the presence of SRF or IRF in macular OCT—may increase the number of injections after surgery and shortens the time to the first injection after surgery [16]. In turn, another study revealed that phacoemulsification in both nAMD eyes characterized by the presence of retinal fluid and nAMD eyes without disease activity leads to significant functional improvement without concomitant deterioration of the macular condition [37]. On the other hand, Choi et al. revealed that the interval between anti-VEGF injections increased 3.4 times after phacoemulsification, while predictive factors associated with a higher frequency of anti-VEGF injections after phacoemulsification were a short exudation-free period and also a longer duration of nAMD, but not the subtype of nAMD, age, diabetes, and the presence of retinal fluid at the time of surgery, or the preoperative injection frequency [31].Therefore, further research is needed to clarify this issue thoroughly.

In our study, we did not observe an exacerbation of nAMD activity in participants undergoing phacoemulsification. There was no negative effect, both anatomically expressed by CRT change or treatment burden manifested as the average number of injections over six months after surgery. Moreover, in our study group, we found improvement in visual function in both the short and long term. Currently, there have been one study demonstrating the effect of cataract surgery on BCVA in nAMD patients treated with anti-VEGF injections over a period of 3–10 years [31]; hence our research fills the observational partial gap and provides a more comprehensive analysis of this issue.

The strengths of our study are the long—54-month—follow-up period for visual outcomes of phacoemulsification in patients with nAMD, as well as the simultaneous evaluation of visual acuity with the frequency of anti-VEGF injections and anatomical changes of the macula monitored with CRT in the six months after surgery. On the other hand, the sample size of our study could have been larger, which would have strengthened the power of our results. However, we believe that our study provides evidences that cataract surgery has a beneficial effect in patients with nAMD treated with anti-VEGF injections—both on visual outcomes in the short and long term, and does not lead to an increase in the frequency of anti-VEGF injections and macular deterioration.

## 5. Conclusions

The results of our study confirm that currently used improved techniques of cataract surgery and nAMD treatment using anti-VEGF agents permit a safe combination of these two therapeutic procedures in patients with co-occurring cataract and well-controlled nAMD, with no signs of MNV activity during two consecutive visits. We have proven that the beneficial effect of phacoemulsification in nAMD patients on visual outcomes is maintained in the short and long term. Cataract surgery in nAMD eyes treated with anti-VEGF injections does not increase the frequency of anti-VEGF injections and does not cause deterioration of macular anatomical parameters.

## Figures and Tables

**Table 1:** Baseline demographic and clinical data of included patients.

Characteristic	Patient’s Data
Sex, n (%)	60 (100)
Female	41 (68.3)
Male	19 (31.7)
Age (years)	
Mean (SD)	77.35 ± 6.41
Median (range)	78 (63–94)
Mean time from diagnosis to anti-VEGF treatment (weeks)	
Mean (SD)	2.95 ± 1.95
Median (range)	2.50 (1–8)
Mean time from start of nAMD treatment to cataract surgery (months)	
Mean (SD)	28.98 ± 20.89
Median (range)	23.50 (6–96)

**Abbreviations:** anti-VEGF, anti-vascular growth endothelial factor; nAMD, neovascular age-related macular degeneration; SD, standard deviation.

**Table 2:** Variability in BCVA in nAMD patients before cataract surgery.

Month before Cataract Surgery	Visual Acuity (logMAR)						
	Mean	Median	Min	Max	Q1	Q3	SD
**6**	0.35	0.30	0.10	0.70	0.20	0.50	0.20
**5**	0.33	0.30	0.00	0.70	0.20	0.40	0.17
**4**	0.34	0.30	0.00	0.70	0.20	0.45	0.19
**3**	0.36	0.30	0.00	0.70	0.20	0.50	0.19
**2**	0.38	0.30	0.00	0.70	0.20	0.50	0.19
**1**	0.42	0.40	0.00	0.70	0.30	0.61	0.20

**Abbreviations**: BCVA, best-corrected visual acuity (logMAR); SD, standard deviation; Q1—first quartile; Q3—third quartile.

**Table 3:** Variability in BCVA in nAMD patients after cataract surgery.

Month after Cataract Surgery	Visual Acuity (logMAR)						
	Mean	Median	Min	Max	Q1	Q3	SD
**1**	0.19	0.10	0.00	0.70	0.00	0.20	0.22
**2**	0.13	0.10	0.00	0.70	0.00	0.10	0.17
**3**	0.13	0.10	0.00	0.70	0.00	0.10	0.17
**4**	0.13	0.10	0.00	0.70	0.00	0.10	0.18
**5**	0.13	0.10	0.00	0.70	0.00	0.10	0.16
**6**	0.13	0.10	0.00	0.70	0.00	0.10	0.16

**Abbreviations**: SD, standard deviation; Q1—first quartile; Q3—third quartile.

**Table 4:** Variability in visual acuity in nAMD patients after cataract surgery.

Months after Cataract Surgery	Visual Acuity (logMAR)						
	Mean	Median	Min	Max	Q1	Q3	SD
**1–6**	0.14	0.10	0.00	1.00	0.00	0.13	0.19
**7–12**	0.14	0.10	0.00	0.70	0.01	0.12	0.16
**13–18**	0.17	0.10	0.00	0.70	0.10	0.20	0.18
**19–24**	0.17	0.10	0.00	0.70	0.10	0.22	0.18
**25–30**	0.16	0.10	0.00	0.70	0.10	0.20	0.16
**31–36**	0.18	0.10	0.00	0.70	0.10	0.22	0.17
**37–42**	0.20	0.10	0.00	0.70	0.10	0.30	0.18
**43–48**	0.25	0.20	0.00	0.70	0.10	0.40	0.21
**49–54**	0.21	0.10	0.00	0.42	0.10	0.40	0.17

**Abbreviations:** Q1, first quartile; Q3, third quartile; SD, standard deviation.

**Table 5:** Correlation between pre- and post-operative mean CRT and post-operative BCVA.

Mean CRT and BCVA Correlation	r	*p* *
Mean CRT during six-month preop and BCVA at day one postop.	−0.27	0.036
Mean CRT during six-month preop and BCVA during six-month postop.	−0.32	0.013
Mean CRT during six-month postop and BCVA at day one postop.	−0.11	0.421
Mean CRT during six-month postop and BCVA during six-month postop.	−0.25	0.058

**Abbreviations:** BCVA, Best-corrected visual acuity (Snellen charts); CRT, central retinal thickness; r—Spearman’s rank correlation test value; *p*—test probability value; * Statistically significant values are marked in bold.

**Table 6:** Time intervals between injections during six-month period before and after cataract surgery.

Time Period	Time Intervals between Injections (Weeks)						
	Mean	Median	Min	Max	Q1	Q3	SD
**Six-month preop.**	9.89	8.69	6.52	26.07	8.69	8.69	4.18
**Six-month postop.**	10.65	8.69	6.52	26.07	8.69	13.04	4.69
	Z = −1.81 *p* = 0.07						

**Abbreviations**: Z—Wilcoxon signed-rank test value; *p*—test probability value.

## Data Availability

All data generated or analyzed during this study are included in this published article.
